# Prognostic value of programmed cell death ligand-1 expression in patients with bladder urothelial carcinoma undergoing radical cystectomy: A meta-analysis

**DOI:** 10.3389/fimmu.2022.986911

**Published:** 2022-09-28

**Authors:** Jindong Zhang, Liangdong Song, Huixuan Zhu, Qinyuan Liu, Delin Wang

**Affiliations:** Department of Urology, the First Affiliated Hospital of Chongqing Medical University, Chongqing, China

**Keywords:** meta-analysis, bladder urothelial carcinoma, programmed cell death ligand-1, prognostic value, radical cystectomy

## Abstract

**Background:**

Radical cystectomy and removal of pelvic lymph nodes (RC-PLND) is a recommended treatment for high-risk non-muscle-invasive and muscle-invasive non-metastatic bladder cancer (BC). However, 50% of patients relapse after RC-PLND. This study aimed to evaluate the effect of programmed cell death ligand-1 (PD-L1) on the prognosis of bladder urothelial carcinoma (BUC) after RC-PLND.

**Methods:**

We present this meta-analysis according to the Preferred Reporting Items for Systematic Review and Meta-Analyses Guidelines. The main outcomes were overall survival (OS), recurrence-free survival (RFS), and cancer-specific survival (CSS) of 3 and 5 years after RC-PLND.

**Results:**

Overall, 11 studies and 1393 BUC cases were included in our meta-analysis. In tumor cells (TCs), the PD-L1 negative group had statistically significant advantage in 5-year OS (risk ratio [RR]: 0.85, 95% confidence interval [CI]: 0.74–0.97, P = 0.02), RFS (RR: 0.76, 95% CI: 0.58–0.99, P = 0.04), and CSS (RR: 0.73, 95% CI: 0.58–0.92, P = 0.009) compared with the PD-L1 positive group. But, no statistically significant difference in 5-year OS and RFS was observed between the PD-L1 negative and positive groups in tumor-infiltrating immune cells.

**Conclusions:**

Our study found that patients with BUC who tested positive for PD-L1 in TCs had a poor prognosis after RC-PLND. PD-1 or PD-L1 inhibitors could be used as a adjuvant medication for patients with BUC after RC-PLND who exhibit PD-L1 overexpression in TCs.

**Systematic review registration:**

https://www.crd.york.ac.uk/prospero/, identifier CRD42022301424.

## Introduction

Bladder cancer (BC) is one of the most common malignant tumors in the world and accounts for more than 500,000 new cases and 200,000 deaths every year (1). Urothelial carcinoma is the most common type of BC and accounts for more than 90% of BC cases (2). According to the latest data of the American Cancer Society in 2022, the incidence and mortality of BC rank second among the tumors of the urinary system (3). Radical cystectomy and removal of pelvic lymph nodes (RC-PLND) is a recommended treatment for high-risk non-muscle-invasive and muscle-invasive non-metastatic BC (4). However, 50% of patients relapse after RC-PLND (5).

Programmed death ligand-1 (PD-L1) expression measured by immunohistochemistry (IHC) is a predictor of the treatment outcome of tumors and has been used in clinical practice. PD-L1 is also the most studied biomarker in tumors (6). Given that BC is an immunogenic tumor (7), blocking the interaction between programmed death-1 (PD-1) and PD-L1 has positively affected BC by restoring T cell-mediated immune response (8). However, PD-L1 expression in tumor cells (TCs) or tumor-infiltrating immune cells (ICs) have different effects on BC prognosis (9–11). Therefore, we need to additional more research and data to obtain a unified result. This biomarker has a high clinical value for the prognosis evaluation and recurrence prevention of patients after radical cystectomy.

This study aimed to evaluate the effect of PD-L1 on the prognosis of bladder urothelial carcinoma (BUC) after RC-PLND. We present this meta-analysis according to the Preferred Reporting Items for Systematic Review and Meta-Analyses Guidelines (12).

## Materials and methods

This study was registered in the International Prospective Register of Systematic Reviews (number: CRD42022301424).

### Search strategy

All studies written in English language and published from PubMed, Embase, and Cochrane Library up to February 15, 2022 were searched independently by two reviewers using the same search formula: (“urothelial carcinoma” [Title/Abstract] OR “bladder carcinoma” OR “bladder cancer” [Title/Abstract] OR “bladder tumor” [Title/Abstract]) AND (“PD-1” [Title/Abstract] OR “programmed death 1” [Title/Abstract] OR “programmed death ligand 1” [Title/Abstract] OR “PD-L1” [Title/Abstract]). The reference lists from published studies were also searched.

### Inclusion and exclusion criteria

Inclusion criteria were as follows: (1) patients with BUC had completed RC-PLND, (2) PD-L1 expression was measured by IHC after RC-PLND, and (3) written in English language. Exclusion criteria were as follows: (1) case report and case series, (2) conference abstract, and (3) incomplete data or duplicated data.

### Data extraction and quality assessment

All records from the three electronic databases were selected using the Endnote software. The data of the included studies were extracted independently by two reviewers using Engauge Digitizer 12.1 software. The quality of included studies was also evaluated. Any disagreements were resolved through discussion with a third reviewer. Newcastle–Ottawa Scale (NOS) (13) was employed to evaluate the quality of all included retrospective studies (**Supplementary Table 1**). The NOS comprises eight items with a maximum of nine scores, with high scores indicating good quality.

### Outcomes of interest

The main outcomes were overall survival (OS), recurrence-free survival (RFS), and cancer-specific survival (CSS) of 3 and 5 years after RC-PLND.

### Statistical analysis

All data analyses were performed using the Review Manager version 5.3 and Stata SE 14.0 tools. The Risk ratio (RR) was used to describe the results of dichotomous variables. p < 0.05 was regarded as statistically significant. Heterogeneities among studies were assessed by heterogeneity (I^2^) and chi-squared (χ^2^) tests. When I^2^ < 50%, a fixed-effect model was used. For I^2^ ≥ 50%, a random-effects model was employed.

## Results

### Selected studies

**Figure 1:**
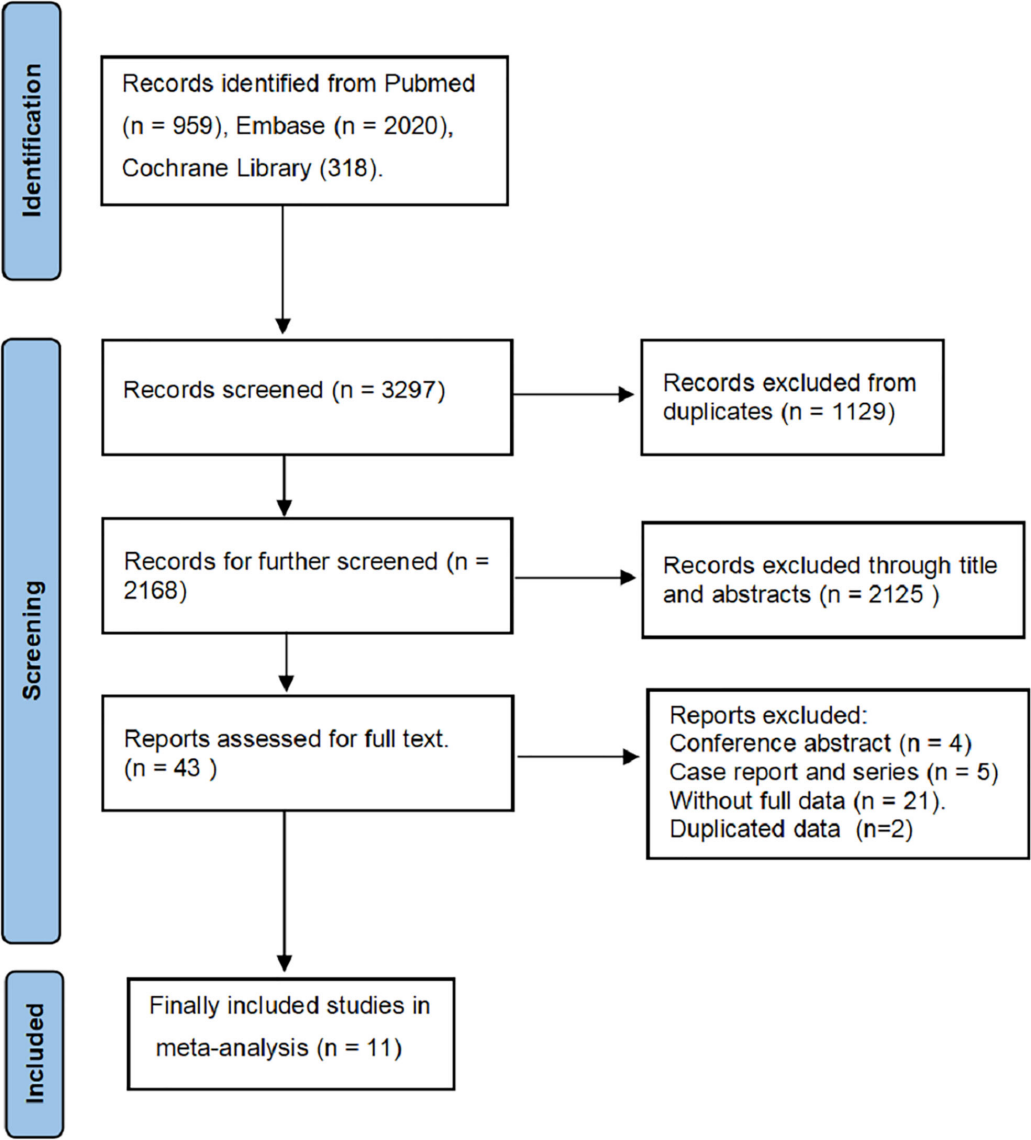
Flow diagram of the selection process of relevant studies.

We searched 3297 records from the three electronic databases (PubMed, Embase, and Cochrane Library). Finally, 11 studies (11, 14–23) and 1393 BUC cases were included in our meta-analysis (**Figure 1**). This study is the first meta-analysis about the prognostic value of PD-L1 expression in patients with BUC after RC-PLND. **Table 1** summarizes the characteristics and quality of all included studies that were published within the past 5 years. The patients were divided into PD-L1 positive group (PD-L1^+^) and PD-L1 negative group (PD-L1^—^) for TCs or ICs.

**Table 1:** Characteristics and quality of included studies.

Author	Year	country	Type of study	Gender Male/Female	Age (year, n, Median (IQR)/(range), Mean ± SD)	TC PD-L1 (n)		IC PD-L1 (n)		NOS Scores
						Positive	Negative	Positive	Negative	
Noro et al. (20)	2017	Japan	Retrospective	82/20	69 (range 43–84)	38	64	–	–	7
Pichler et al. (18)	2018	Austria	Retrospective	62/21	69 (range 36–87)	33	50	51	30	7
Wang et al. (15)	2019	China	Retrospective	214/34	≤ 60, 101; > 60, 147	58	190	136	112	8
Eckstein et al. (21)	2018	Germany	Retrospective	–	70.2 (range 41.3–90.8)	–	–	29	59	6
Toren et al. (16)	2020	Canada	Retrospective	154/44	67.95 (61.13 - 75.75)	–	–	95	80	8
Rubino et al. (17)	2021	USA	Retrospective	98/39	65 (range 33–84)	52	64	–	–	8
Murakami et al. (19)	2021	Japan	Retrospective	80/17	66 (41−80)	14	60	34	63	6
Tural et al. (11)	2021	Turkey	Retrospective	56/5	≤ 64, 34; > 64, 27	9	52	10	51	7
Nechifor-Boil et al. (23)	2021	Switzerland	Retrospective	57/12	67.35 ± 9.98	28	41	–	–	8
Lee et al. (22)	2021	South Korea	Retrospective	75/17	< 72, 45; ≥ 72, 47	–	–	50	42	7
Horiguchi et al. (14)	2021	Japan	Retrospective	199/63	69 (62-75)	92	170	–	–	7

IQR interquartile range, SD standard deviation, TC tumor cell, IC tumor-infiltrating immune cells, PD-L1 programmed death ligand-1, NOS newcastle-ottawa scale.

### OS

**Figure 2:**
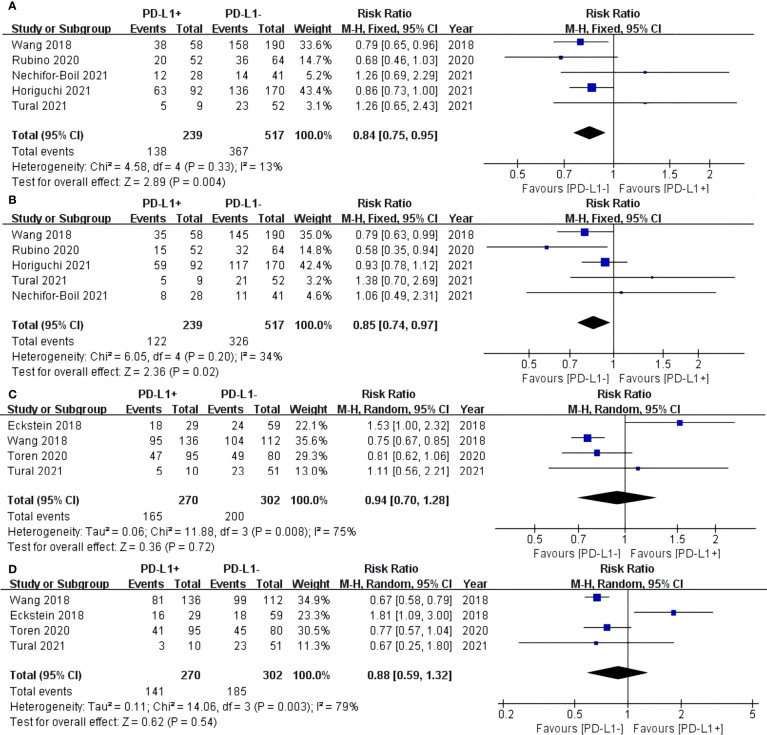
Forest plot of overall survival (OS) between the PD-L1 negative and positive groups in tumor cells (TCs): OS after surgery 3 years **(A)** and 5 years **(B)**. Forest plot of OS between two group in tumor-infiltrating immune cells (ICs): OS after surgery 3 years **(C)** and 5 years **(D)**.

For TCs, the PD-L1 negative group had a statistically significant better OS of 3 years (RR: 0.84, 95% CI: 0.75–0.95, P = 0.004) (**Figure 2A**) and 5 years (RR: 0.85, 95% CI: 0.74–0.97, P = 0.02) (**Figure 2B**) compared with the PD-L1 positive group. For ICs, no statistically significant difference in 3-year (RR: 0.94, 95% CI: 0.70–1.28, P = 0.72) and 5-year (RR: 0.88, 95% CI: 0.59–1.32, P = 0.54) OS was found between the PD-L1 negative and positive groups (**Figures 2C, D**).

### RFS

**Figure 3:**
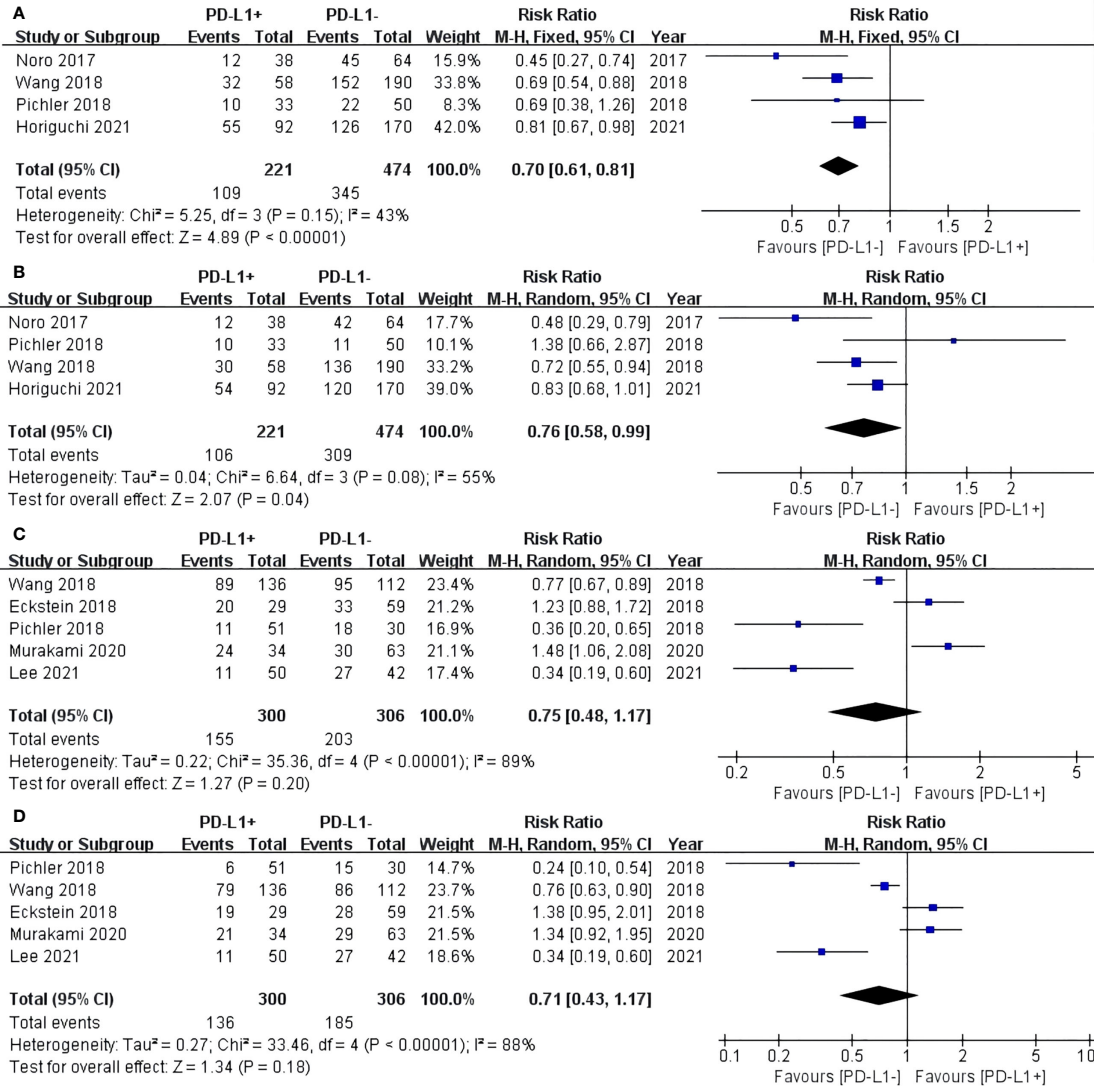
Forest plot of recurrence-free survival (RFS) between two group in TCs: RFS after surgery 3 years **(A)** and 5 years **(B)**. Forest plot of RFS between two group in ICs: RFS after surgery 3 years **(C)** and 5 years **(D)**.

For TCs, the PD-L1 negative group had a statistically significant advantage in 3-year (RR: 0.70, 95% CI: 0.61–0.81, P < 0.00001) (**Figure 3A**) and 5-year (RR: 0.76, 95% CI: 0.58–0.99, P = 0.04) RFS after RC-PLND (**Figure 3B**). For ICs, no statistically significant difference in 3-year (RR: 0.75, 95% CI: 0.48–1.17, P = 0.20) and 5-year (RR: 0.71, 95% CI: 0.43–1.17, P = 0.18) RFS was found between the two groups (**Figures 3C, D**).

### CSS

**Figure 4:**
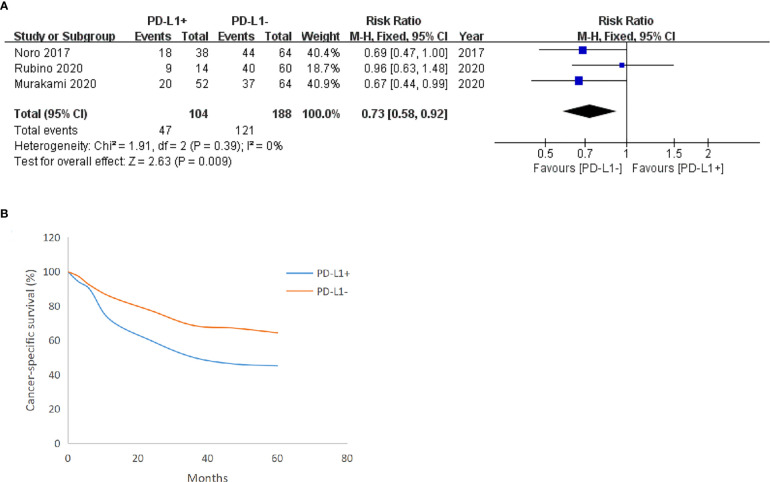
Forest plot of cancer-specific survival (CSS) between two group in TCs after surgery 5 years **(A)**, CSS trend chart between the group in TCs **(B)**.

Three included studies reported the CSS of patients with BUC after RC-PLND with PD-L1 detected on TCs. For TCs, the PD-L1 negative group had a statistically significant advantage in 5-year (RR: 0.73, 95% CI: 0.58–0.92, P = 0.009) CSS (**Figure 4**).

### Sensitivity analysis

**Figure 5:**
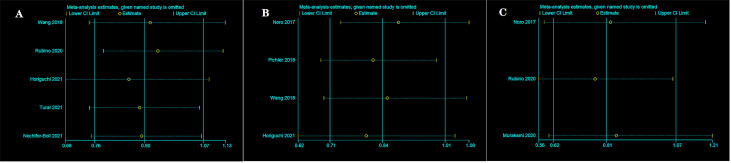
Sensitivity analysis between two group in TCs after surgery 5 years. OS **(A)**, RFS **(B)**, and CSS **(C)**.

We conducted the sensitivity analysis of OS (**Figure 5A**), RFS (**Figure 5B**), and CSS (**Figure 5C**) for PD-L1 expression on TCs at 5 years after surgery by separately removing each study and then merging the effect quantity. The results showed no remarkable changes in the overall results after the exclusion of each study from the main analysis.

### Publication bias

**Figure 6:**
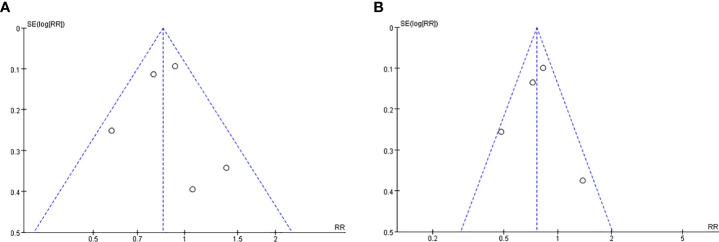
Funnel plot between two group in TCs after surgery 5 years. OS **(A)** and RFS **(B)**.

We constructed the funnel plot of OS (**Figure 6A**) and RFS (**Figure 6B**) for PD-L1 expression in TCs at 5 years after surgery. The funnel plot was basically symmetrical, indicating the low probability of publication bias.

## Discussion

The high recurrence rate after radical cystectomy for BC has become a problem for doctors and patients. This study is the first meta-analysis of retrospective cohort studies for patients with BUC after radical cystectomy with long clinical follow-up time. The results showed the prognostic value of PD-L1 staining in bladder tumors.

In TCs, the PD-L1 negative group had statistically significant advantage in 5-year OS (63.1% vs. 51.0%), RFS (65.2% vs. 48.0%), and CSS (64.4% vs. 45.2%) compared with the PD-L1 positive group. This finding indicated that the prognosis of PD-L1 positive patients is worse after radical surgery due to the ability of PD-L1 to promote tumor immune escape (24). Overexpressed PD-L1 on TCs binds to PD-1 on ICs, and the TCR-signaling cascade is counteracted by SHP-2 phosphorylation. Finally, the activation of T cells is impaired (25).

However, no statistically significant difference in 5-year OS and RFS was observed between the PD-L1 negative and positive groups in ICs. This finding confirmed that the PD-L1 expression is more correlated with patient prognosis in TCs than in ICs (26).

Different results for PD-L1 expression have been reported in TCs or ICs removed by radical cystectomy (14, 17–19) due to the insufficient population included in single-center studies or the use of different PD-L1 positive determination methods that may interfere with the results from no correlation to poor prognosis. Our meta-analysis included many patients and studies conducted in recent years. The current results showed that patients with BUC who tested positive for PD-L1 in TCs had a poor prognosis after RC-PLND. Meanwhile, the PD-L1 expression in ICs had no statistical significance in the prognosis of patients with BUC after RC-PLND. Therefore, PD-1 or PD-L1 inhibitors can be used as a postoperative medication for patients with BUC after RC-PLND who exhibit PD-L1 overexpression in their TCs.

Shen et al. reported that compared with traditional drugs, PD-1 or PD-L1 inhibitors prolong the OS time of patients as second or later lines of treatment in advanced solid tumors with PD-L1 positive or negative expression (27). Funt et al. reported that neoadjuvant atezolizumab with gemcitabine and cisplatin chemotherapy effectively reduced the pathological stage of patients with muscle-invasive BC prior to RC-PLND and improved their OS and RFS rates (28). PD-1 or PD-L1 inhibitors have been used in advanced BC or neoadjuvant BC treatment and have achieved good results (27, 28). We expect that this regime will be utilized for patients who underwent radical cystectomy as a preventive rather than a salvage treatment. Molecular pathology and metabolomics have driven efforts to classify BC into subtypes, which could be potentially valuable in predictions of clinical outcomes and benefit to early diagnosis and treatment (29).

Our study had several limitations. First, no RCTs were included. Second, further subgroup analysis according to clinical or pathological stages was not conducted due to lack of data. Third, when a patient relapses after surgery, the use of different treatment methods may affect the patient’s OS and CSS. However, our results are still credible and have passed the heterogeneity test, publication bias test, and sensitivity analysis.

## Conclusion

Our meta-analysis found that patients with BUC who tested positive for PD-L1 in TCs had a poor prognosis after RC-PLND. Meanwhile, PD-L1 expression in ICs had no statistical significance in the prognosis of patients after RC-PLND. PD-1 or PD-L1 inhibitors could be used as a adjuvant medication for patients with BUC after RC-PLND who exhibit PD-L1 overexpression in TCs; however, randomized controlled trials are needed for further verification.

## Data availability statement

The original contributions presented in the study are included in the article/**Supplementary Material**. Further inquiries can be directed to the corresponding author.

## Author contributions

JZ and LS contributed to the conception and design this study. JZ, HZ and QL were responsible for the development of the methodology and data interpretation. JZ analyzed and interpreted the data. JZ wrote the paper. DW revised the paper. All authors contributed to the article and approved the submitted version.

## Funding

This work was supported by grants from the Natural Science Foundation of Chongqing (cstc2019jcyj-msxmX0732), and Chongqing Science and Technology Commission and Technology Commission (cstc2017shms-zdyf0319).

## Conflict of interest

The authors declare that the research was conducted in the absence of any commercial or financial relationships that could be construed as a potential conflict of interest.

## Publisher’s note

All claims expressed in this article are solely those of the authors and do not necessarily represent those of their affiliated organizations, or those of the publisher, the editors and the reviewers. Any product that may be evaluated in this article, or claim that may be made by its manufacturer, is not guaranteed or endorsed by the publisher.

## References

[1] Tran L Xiao JF Agarwal N Duex JE Theodorescu D. Advances in bladder cancer biology and therapy. Nat Rev Cancer. (2021) 21(2):104–21. doi: 10.1038/s41568-020-00313-1 PMC10112195 33268841

[2] Heath E Rosenberg JE. The biology and rationale of targeting nectin-4 in urothelial carcinoma. Nat Rev Urol. (2021) 18(2):93–103. doi: 10.1038/s41585-020-00394-5 33239713

[3] Siegel RL Miller KD Fuchs HE Jemal A. Cancer statistics, 2022. CA Cancer J Clin (2022) 72(1):7–33. doi: 10.3322/caac.21708 35020204

[4] Witjes JA Bruins HM Cathomas R Compérat EM Cowan NC Gakis G. European Association of urology guidelines on muscle-invasive and metastatic bladder cancer: Summary of the 2020 guidelines. Eur Urol. (2021) 79(1):82–104. doi: 10.1016/j.eururo.2020.03.055 32360052

[5] Yoo SH Kim H Kwak C Kim HH Jung JH Ku JH. Late recurrence of bladder cancer following radical cystectomy: Characteristics and outcomes. Urol Int (2019) 103(3):291–6. doi: 10.1159/000502656 31461728

[6] Savic Prince S Bubendorf L. Predictive potential and need for standardization of PD-L1 immunohistochemistry. Virchows Arch (2019) 474(4):475–84. doi: 10.1007/s00428-018-2445-7 30173280

[7] van Wilpe S Gerretsen ECF van der Heijden AG de Vries IJM Gerritsen WR Mehra N. Prognostic and predictive value of tumor-infiltrating immune cells in urothelial cancer of the bladder. Cancers (Basel). (2020) 12(9):2692. doi: 10.3390/cancers12092692 PMC7565173 32967190

[8] Thomas Powles T Eder JP Fine GD Braiteh FS Loriot Y Cristina C. MPDL3280A (anti-PD-L1) treatment leads to clinical activity in metastatic bladder cancer. Nature. (2014) 515(7528):558–62. doi: 10.1038/nature13904 25428503

[9] Hahn AW Sirohi D Agarwal N. The role of PD-L1 testing in advanced genitourinary malignancies. Eur Urol Focus. (2020) 6(1):11–3. doi: 10.1016/j.euf.2019.03.003 30872123

[10] Bellmunt J Mullane SA Werner L Fay AP Callea M Leow JJ. Association of PD-L1 expression on tumor-infiltrating mononuclear cells and overall survival in patients with urothelial carcinoma. Ann Oncol (2015) 26(4):812–7. doi: 10.1093/annonc/mdv009 25600565

[11] Tural D Akar E Baytekin HF Canoglu D Yilmaz M Tugcu V. Relationship between survival outcomes and microsatellite instability, tumor infiltrating lymphocytes and programmed cell death ligand-1 expression in patients with bladder cancer and radical cystectomy. J BUON. (2021) 26(5):2117–25. 34761625

[12] Moher D Shamseer L Clarke M Ghersi D Liberati A Petticrew M. Preferred reporting items for systematic review and meta-analysis protocols (PRISMA-p) 2015 statement. Syst Rev (2015) 4:1. doi: 10.1186/2046-4053-4-1 25554246 PMC4320440

[13] Stang A. Critical evaluation of the Newcastle-Ottawa scale for the assessment of the quality of nonrandomized studies in meta-analyses. Eur J Epidemiol. (2010) 25:603–5. doi: 10.1007/s10654-010-9491-z 20652370

[14] Horiguchi H Hatakeyama S Yoneyama T Yoneyama MS Tanaka T Fujita N. Prognostic significance of the Ki67 index and programmed death-ligand 1 expression after radical cystectomy in patients with muscle-invasive bladder cancer. Urol Oncol (2021) 39(4):238. e9–238. e17. doi: 10.1016/j.urolonc.2020.11.029 33308976

[15] Wang B Pan W Yang M Yang W He W Chen X. Programmed death ligand-1 is associated with tumor infiltrating lymphocytes and poorer survival in urothelial cell carcinoma of the bladder. Cancer Sci (2019) 110(2):489–98. doi: 10.1111/cas.13887 PMC6361576 30548363

[16] Toren p Brisson H Simonyan D Hovington H Lacombe L Bergeron A. Androgen receptor and immune cell PD-L1 expression in bladder tumors predicts disease recurrence and survival. World J Urol. (2021) 39(5):1549–58. doi: 10.1007/s00345-020-03358-x 32676741

[17] Rubino S Kim Y Zhou J Dhilon J Li R Spiess P. Positive ki-67 and PD-L1 expression in post-neoadjuvant chemotherapy muscle-invasive bladder cancer is associated with shorter overall survival: A retrospective study. World J Urol. (2021) 39(5):1539–47. doi: 10.1007/s00345-020-03342-5 PMC10091226 32656671

[18] Pichler R Fritz J Lackner F Sprung S Brunner A Horninger W. Prognostic value of testing PD-L1 expression after radical cystectomy in high-risk patients. Clin Genitourin Cancer (2018) 16(5):e1015–24. doi: 10.1016/j.clgc.2018.05.015 29960831

[19] Murakami Y Matsumoto K Shimizu Y Ikeda M Amano N Shimura S. PD-L1 expression in tumor-infiltrating lymphocytes (TILs) as an independent predictor of prognosis in patients with pN0 bladder cancer undergoing radical cystectomy. Urol Oncol (2021) 39(3):195. doi: 10.1016/j.urolonc.2020.09.034 33071109

[20] Noro D Hatakeyama S Yoneyama T Hashimoto Y Koie T Kawaguchi T. Post-chemotherapy PD-L1 expression correlates with clinical outcomes in Japanese bladder cancer patients treated with total cystectomy. Med Oncol (2017) 34(6):117. doi: 10.1007/s12032-017-0977-3 28500617

[21] Eckstein M Wirtz RM Pfannstil C Wach S Stoehr R Breyer J. A multicenter round robin test of PD-L1 expression assessment in urothelial bladder cancer by immunohistochemistry and RT-qPCR with emphasis on prognosis prediction after radical cystectomy. Oncotarget. (2018) 9(19):15001–14. doi: 10.18632/oncotarget.24531 PMC5871092 29599921

[22] Lee CU Lee DH Song W. Prognostic role of programmed death ligand-1 on tumor-infiltrating immune cells in “High-risk” patients following radical cystectomy: A retrospective cohort study. Front Oncol (2021) 11:706503. doi: 10.3389/fonc.2021.706503 34490106 PMC8417560

[23] Nechifor-Boilă IA Loghin A Nechifor-Boilă A Decaussin-Petrucci M Voidăzan S Chibelean BC. PD-L1 expression in muscle invasive urothelial carcinomas as assessed *via* immunohistochemistry: Correlations with specific clinical and pathological features, with emphasis on prognosis after radical cystectomy. Life (Basel). (2021) 11(5):404. doi: 10.3390/life11050404 33925149 PMC8146852

[24] Jiang X Wang J Deng X Xiong F Ge J Xiang B. Role of the tumor microenvironment in PD-L1/PD-1-mediated tumor immune escape. Mol Cancer. (2019) 18(1):10. doi: 10.1186/s12943-018-0928-4 30646912 PMC6332843

[25] Yi M Niu M Xu L Luo S Wu K. Regulation of PD-L1 expression in the tumor microenvironment. J Hematol Oncol (2021) 14(1):10. doi: 10.1186/s13045-020-01027-5 33413496 PMC7792099

[26] Taube JM Klein A Brahmer JR Xu H Pan X Kim JH. Association of PD-1, PD-1 ligands, and other features of the tumor immune microenvironment with response to anti-PD-1 therapy. Clin Cancer Res (2014) 20(19):5064–74. doi: 10.1158/1078-0432.CCR-13-3271 PMC4185001 24714771

[27] Shen X Zhao B. Efficacy of PD-1 or PD-L1 inhibitors and PD-L1 expression status in cancer: Meta-analysis. BMJ (2018) 362:k3529. doi: 10.1136/bmj.k3529 30201790 PMC6129950

[28] Funt SA Lattanzi M Whiting K Al-Ahmadie H Quinlan C Teo MY. Neoadjuvant atezolizumab with gemcitabine and cisplatin in patients with muscle-invasive bladder cancer: A multicenter, single-arm, phase II trial. J Clin Oncol (2022), 40(12):1313–22. doi: 10.1200/JCO.21.01485 PMC9797229 35089812

[29] Di Meo NA Loizzo D Pandolfo SD Autorino R Ferro M Porta C. Metabolomic approaches for detection and identification of biomarkers and altered pathways in bladder cancer. Int J Mol Sci (2022) 23:4173. doi: 10.3390/ijms23084173 35456991 PMC9030452

