# Supplementary material for: Prognostic value of programmed cell death ligand-1 expression in patients with bladder urothelial carcinoma undergoing radical cystectomy: A meta-analysis
Source: Front Immunol. 2022 Sep 28;13:986911. doi: 10.3389/fimmu.2022.986911 (PMC9554211; doi:10.3389/fimmu.2022.986911)
Supplement: Supplementary file 1 [file Table_1.doc]

| **Author** | **Year** | **Selection** | | | | **Comparability** | | **Outcome** | | | **Total score** |
| --- | --- | --- | --- | --- | --- | --- | --- | --- | --- | --- | --- |
| Noro et al. | 2017 | **🟑** | **🟑** | **🟑** | - | **🟑** | - | **🟑** | **🟑** | **🟑** | 7 |
| Pichler et al. | 2018 | **🟑** | **🟑** | **🟑** | - | **🟑** | - | **🟑** | **🟑** | **🟑** | 7 |
| Wang et al. | 2018 | **🟑** | **🟑** | **🟑** | - | **🟑** | **🟑** | **🟑** | **🟑** | **🟑** | 8 |
| Eckstein et al. | 2018 | **🟑** | **🟑** | **🟑** | - | **🟑** | - | **🟑** | - | **🟑** | 6 |
| Toren et al. | 2020 | **🟑** | **🟑** | **🟑** | - | **🟑** | **🟑** | **🟑** | **🟑** | **🟑** | 8 |
| Rubino et al. | 2020 | **🟑** | **🟑** | **🟑** | - | **🟑** | **🟑** | **🟑** | **🟑** | **🟑** | 8 |
| Murakami et al. | 2020 | **🟑** | **🟑** | **🟑** | - | **🟑** | - | **🟑** | - | **🟑** | 6 |
| Tural et al. | 2021 | **🟑** | **🟑** | **🟑** | - | **🟑** | - | **🟑** | **🟑** | **🟑** | 7 |
| Nechifor-Boil et al. | 2021 | **🟑** | **🟑** | **🟑** | - | **🟑** | **🟑** | **🟑** | **🟑** | **🟑** | 8 |
| Lee et al. | 2021 | **🟑** | **🟑** | **🟑** | - | **🟑** | - | **🟑** | **🟑** | **🟑** | 7 |
| Horiguchi et al. | 2021 | **🟑** | **🟑** | **🟑** | - | **🟑** | - | **🟑** | **🟑** | **🟑** | 7 |

**Supplementary Table. 1 Quality assessments of included studies based on** **Newcastle Ottawa Scale**

**Selection 0-4 🟑**

1) Representativeness of the exposed cohort

2) Selection of the non exposed cohort

3) Ascertainment of exposure

4) Demonstration that outcome of interest was not present at start of study

**Comparability 0-2 🟑**

Comparability of cohorts on the basis of the design or analysis

**Outcome 0-3 🟑**

1) Assessment of outcome

2) Was follow-up long enough for outcomes to occur

3) Adequacy of follow up of cohorts
